# SHOX deficiency in children with growth impairment: evaluation of known and new auxological and radiological indicators

**DOI:** 10.1186/s13052-020-00927-z

**Published:** 2020-11-03

**Authors:** Silvia Vannelli, Maria Baffico, Raffaele Buganza, Francesca Verna, Giulia Vinci, Daniele Tessaris, Gianpaolo Di Rosa, Alberto Borraccino, Luisa de Sanctis

**Affiliations:** 1 Pediatric Endocrinology, Regina Margherita Children’s Hospital, Turin, Italy; 2 Department of Public Health and Pediatric Sciences, University of Turin, Turin, Italy; 3 Laboratory of Human Genetics, Galliera Hospitals, Genoa, Italy; 4 Postgraduate School of Pediatrics, University of Turin, Turin, Italy; 5 Pediatric Radiology, Regina Margherita Children’s Hospital, Turin, Italy

**Keywords:** SHOX, Growth impairment, Convexity of distal radial metaphysis

## Abstract

**Background:**

The phenotypic features of SHOX deficiency (SHOX-D) are highly variable and can be very mild, especially in young children. The aim of this retrospective study was to evaluate auxological and radiological indicators that could be predictive of SHOX-D in children.

**Methods:**

Molecular analysis of the SHOX gene was performed in 296 subjects with growth impairment or skeletal disproportion, without alternative diagnosis. Auxological variables and radiographs of the hand, wrist and forearm were evaluated.

**Results:**

SHOX mutations (88% inherited, 12% de novo) were identified in 52 subjects. The most predictive auxological indicators of SHOX-D were an increased sitting height/height ratio and a decreased arm span/height ratio. The convexity of distal radial metaphysis at X-ray, not yet reported in literature, was also found to be predictive of SHOX-D. In young children, stratification of data by bone age also highlighted ulnar tilt, lucency of the ulnar border of the distal radius and enlarged radius as the radiological signs most related to SHOX-D .

**Conclusions:**

In this study, the analysis of auxological and radiological indicators in SHOX-D children allowed to identify an additional early radiological sign and underlines the importance of family auxological evaluation.

## Background

The defect of the short-stature homeobox-containing (SHOX) gene, located on the pseudoautosomal region (PAR1) of the X and Y chromosomes, is the most frequent cause of monogenic short stature [1, 2]. SHOX is involved in pre- and postnatal skeletal development as it regulates the differentiation and apoptosis of chondrocytes in the epiphyseal growth plate [1, 3]. SHOX deficiency (SHOX-D) causes short stature with a highly variable phenotype, ranging from an extreme dwarfism, with mesomelia and limb deformity as seen in Langer syndrome (caused by two defective or absent SHOX alleles) to a disproportionate short stature with mesomelia known as Léri–Weill dyschondrosteosis (caused by defective or loss of a single SHOX allele), to apparently idiopathic short stature (ISS) with no other obvious clinical signs [4]. The frequently mild phenotypic expression renders difficult to define when to proceed with SHOX molecular analysis in children with growth impairment. The selection should be driven by specific anthropometric measurements, family history, presence of dysmorphisms or peculiar radiological signs in the hand/wrist and forearms X-ray. Rappold et al. suggested a scoring system based on eight clinical criteria [4] and Binder proposed a diagnostic algorithm based on clinical and radiological criteria [5]. The most reported X-ray abnormalities are triangularization of the distal radial epiphysis, pyramidalization of the distal carpal row, lucency of the ulnar border of the distal radius and bowing of the radius [5–8] . However, the diagnosis of SHOX-D is particularly challenging in preschool age, when skeletal mesomelic disproportions and Madelung deformity may still be absent [5]. Literature data indicate that recombinant human Growth Hormone (rhGH) treatment improves the growth pattern [9] in SHOX-D, particularly when it is started in early childhood [10]; therefore, it becomes essential to reach an early definitive diagnosis to begin an early rhGH treatment. The aim of this retrospective study was to evaluate the association between different auxological and radiological indicators and the presence of SHOX haploinsufficiency.

## Patients and methods

### Patients

Two hundred ninety-six children (180 males and 116 females, aged 3–18 years) were enrolled at the Auxological Center of Regina Margherita Children’s Hospital in Turin, from January 2011 to January 2018. Patients with at least one of the following parameters were included: short stature (height < 3rd age- and sex- related percentile), sitting height/height ratio (SH/H) > 2 SDS, predicted adult height *more than 2 SDS below* the mid-parental height, height velocity < 25th age- and sex- related percentile for more than one year. Exclusion criteria were chronic disease, other already defined genetic or hormonal disease, treatment with drugs that affect growth, malnutrition and psychosocial disorders.

### Clinical assessment

Height was measured to the nearest 0.1 cm with calibrated stadiometer according to Cameron’s method [11]. Body weight was evaluated to the nearest 0.1 kg using a calibrated balance scale. SH, SH/H (expressed as SDS), body mass index (BMI), arm span, arm spam/ height ratio and pubertal stages were assessed. Parents’ height were measured with calibrated stadiometer and mid-parental height was calculated with Tanner’s formula [12]. Anthropometric measures were analyzed according to the following references: Tanner et al. for stature, weight and height velocity [13, 14], Fredriks et al. for SH:H [15]; Cacciari et al. for BMI [16]; INeS charts for neonatal weight and length [17].

### Radiological evaluation

**Fig. 1:**
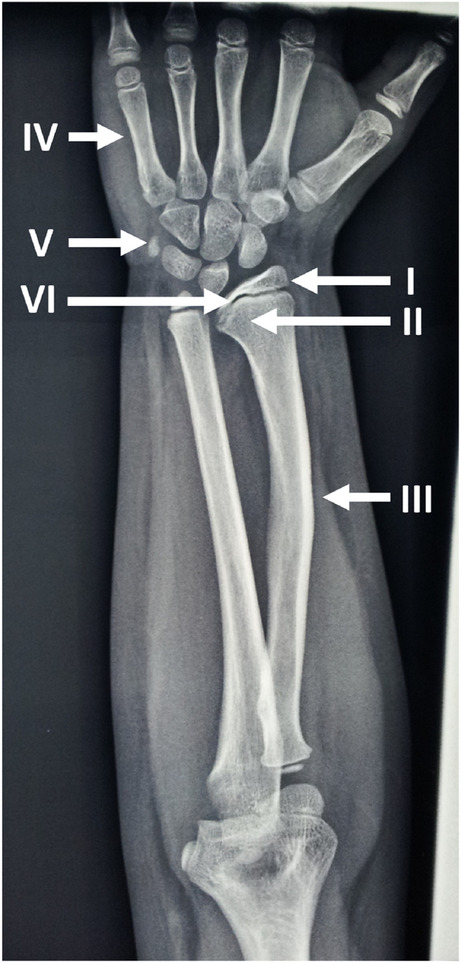
Radiological features in SHOX deficiency. I: triangularization of the distal radial epiphysis, II: lucency of the ulnar border of the distal radius, III: enlarged diaphysis of radius plus bowing of radius, IV: Short 4th and 5th metacarpal, V: pyramidalization of the carpal row, VI: convexity of distal radial metaphysis

An expert radiologist and two trained clinicians examined 230 X-rays (115 hand, wrist and forearm, 109 hand and wrist, 6 forearm X-rays), focusing on the following radiological indicators of SHOX-D [5–8, 18, 19] (Fig. 1): triangularization index of the distal radial epiphysis (maximum lateral length/ central length ratio) in females with bone age > 10.5 and males with bone age > 11.5 years, carpal angle (to evaluate the pyramidalization of the carpal row), lucency of the ulnar border of the distal radius, ulnar tilt (reported as percentage of cases with value > 33° [19]), distal radio-ulnar physeal disparity, short 4th metacarpal, short 5th metacarpal. Bowing of the radius, presence of enlarged diaphysis of the radius and association of both signs were also evaluate, as well as the shape of the distal radial metaphysis (reported as percentage of cases with convex shape). Bone age was assessed according to the TW2-RUS quantitative method [20].

### Molecular analysis

Genomic DNA was extracted from peripheral blood lymphocytes. MLPA (Multiplex Ligation-dependent Probe Amplification) analysis for deletions and duplications was carried out with the commercial kit SALSA PO18-F1 SHOX (MCR Holland, Amsterdam, Netherlands) in accordance to the manufacturer’s recommendations. Specific probes were used for each exon within the SHOX gene and for some regulatory gene sequences in PAR1. Point mutations, microinsertion, microdeletion in exons 2,3,4,5,6,6a and in the flanking regions of SHOX gene were searched by direct sequencing, using DNA Analyzer Applied Biosystems. We included in the study only pathogenic or likely pathogenic variants, according to the ACMG guidelines [21].

### Statistical analysis

T-test was used to evaluate the differences between groups. For the analysis of categorical and dichotomous variables the X^2^ test was applied. We reported qualitative variables as percentage and quantitative variables as mean ± SD. For all tests, *p* < 0.05 was considered statistically significant. The software used for statistical elaboration was STATA, version 8.

## Results

Heterozygous SHOX mutations were identified in 52 of the 296 screened participants (17.6%). The prevalence of SHOX-D was 19.7% in the group of subjects with ISS (6.7% in children with ISS and normal SH/H, 28.3% in those with ISS and SH/H > 2 SDS) and 60% in patients with Léri–Weill (LW) phenotype; 88% of variants were inherited from one parent, 22% were de novo.

The main clinical features of the patient groups with and without SHOX-D (SHOX-D+ and SHOX-D-) are summarized in Table 1. SHOX-D+ had higher SH/H, lower arm span/ height ratio and higher bone age.

**Table 1:** Clinical and auxological features of the whole population

	SHOX D+	SHOX D-	p value
Subjects, n. of patients	52	244	
Males/Females, %	50/50	63/37	.055
Age at diagnosis, years	10.9 (3.7)	10.4 (2.6)	.305
Height, SDS	−1.58 (0.9)	−1.58 (0.6)	.455
Sitting height/height, SDS	+ 2.51 (1.2)	+ 1.91 (0.9)	< .001
Arm spam/height	0.979 (0.04)	0.989 (0.02)	.027
BMI, SDS	0.1 (0.3)	0.1 (0.4)	.586
Bone age, years	10.4 (3.2)	9.2 (2.6)	.007
Neonatal length, SDS	−0.8 (1.1)	−0.8 (1.0)	.996
Neonatal weight, SDS	−0.5 (1.0)	−0.6 (1.0)	.458

Mean (SD) values are reported

The radiological findings are reported in Table 2 and Fig. 1. SHOX-D+ and SHOX-D- subjects differed significantly for triangularization index (TI), lucency of the ulnar border of the distal radius, ulnar tilt, short 4th metacarpal, convexity of distal radial metaphysis, enlarged diaphysis of the radius and the combined criterion of enlarged diaphysis plus bowing of radius. Gender- stratified analyses showed that all those signs mainteined significant differences only in females. Stratifying the data for bone age (Table 3), in the younger group lucency, ulnar tilt, enlarged diaphysis of the radius, convexity of distal radial metaphysis and the combined criterion of enlarged diaphysis plus bowing of the radius were significantly different between SHOX-D+ and SHOX-D- subjects, while in the older group the difference was significant for TI, ulnar tilt and convexity of distal radial metaphysis.

**Table 2:** Radiological signs of the whole population

	SHOX D+	SHOX D-	p value
Triangularization index^a^	9.5 (4.2)	6.8 (2.3)	.002
Carpal angle, degrees	128.2 (9.9)	130.3 (8.4)	.17
Lucency of the ulnar border of the distal radius, %	62.2	34.8	.001
Ulnar tilt, %	20	0.5	.000
Radio-ulnar physeal disparity, mm	2.7 (1.6)	2.6 (1.5)	.7
Short 4th metacarpal, %	25.0	12.1	.03
Short 5th metacarpal, %	2.3	2.7	.7
Bowing of the radius, %	90	81.2	.3
Enlarged diaphysis of radius, %	80	45.8	.005
Enlarged diaphysis of radius plus bowing of radius, %	75	41.7	.007
Convexity of distal radial metaphysis, %	62.2	28.6	.000

^a^This sign has been evaluated in females subjects with bone age > 10.5 years and males > 11.5 years. Mean (SD) values are reported

**Table 3:** Radiological signs stratified by bone age

	F < 10.5 years, M < 11.5 years		p value	F > 10.5 years, M > 11.5 years		***p*** value
	SHOX D+	SHOX D -		SHOX D+	SHOX D -	
Triangularization index	–	–	–	9.5 (4.2)	6.8 (2.3)	.002
Carpal angle, degrees	129.0 (10.0)	130.3 (8.4)	.6	127.1 (10.4)	131.0 (8.4)	.1
Lucency of the ulnar border of the distal radius, %	52.2	28.7	.03	73.7	50	.08
Ulnar tilt, %	17.4	0.7	.000	21.1	0	.002
Radio-ulnar physeal disparity, mm	2.4 (1.4)	2.5 (1.5)	.8	3.3 (1.6)	2.9 (1.7)	.4
Short 4th metacarpal, %	13.0	10.9	.8	36.9	16.7	.08
Short 5th metacarpal, %	0	1.5	.6	5.3	7.1	.8
Bowing of the radius, %	91.7	80–5	.35	85.7	82.3	.8
Enlarged diaphysis of radius, %	83.3	48.1	.02	71.4	35.3	.1
Enlarged diaphysis of radius plus bowing of radius, %	75.0	42.9	.04	71.4	35.3	.1
Convexity of distal radial metaphysis, %	56.2	23.4	.001	73.7	45.2	.04

*F* females, *M* males. Mean (SD) values are reported

In parents with SHOX-D, final height was − 1.9 SDS (range − 3.83/+ 0.25 SDS) in females and − 1.2 SDS (− 3.66/ + 0.58 SDS) in males and skeletal disproportion was present in 91 and 75% in females and males, respectively.

## Discussion

Since the effects of SHOX-D are highly variable and can even be mild, particularly in early childhood, we decided to include subjects with different types of growth impairment in this study. Among the LW phenotype patients in our series, the 60% prevalence of SHOX-D is consistent with literature data (50–90%) [5], although the distinction between LWD and non-LWD is somewhat arbitrary considering the continuum of the clinical phenotype in SHOX-D [4]. In the group of subjects with apparently ISS, the prevalence of SHOX-D was higher in subjects with skeletal disproportion than in those without disproportion (28.3% vs 6.7%), confirming previous studies reporting SHOX mutations in 1–22% of the ISS [1], with the highest prevalence in disproportionate ISS [22]. Finally, it is noteworthy that, within the series analyzed for mutations in the SHOX gene, 57.7% of the subjects found to be mutated had a stature greater than the third percentile at the diagnosis.

In our study, SHOX-D subjects had the expected skeletal disproportion, with higher SH/H and lower arm span/height ratio. Furthermore, the 88% of SHOX mutations were inherited from parents, who had disproportions in a higher percentage of cases. Thus, the auxological evaluation of parents is of utmost importance since it allows to detect familial disproportionate short stature and additional signs of SHOX-D that may be absent in children.

The radiological analysis of the hand, wrist and forearm by experts allowed to highlight the convexity of the distal radial metaphysis as a new sign related to SHOX-D, with a prevalence of 62%. In the SHOX-D+ group, the end of the metaphysis was more frequently convex, with an increased curvature of the growth plate, more pronounced in the medial half. It may therefore be useful to look for this further simple sign prior to growth plate fusion as it may be related to the other growth plate abnormalities in SHOX-D [3, 23, 24].

In the mutated series, the typical alteration in lucency of the distal ulnar border of the radius was confirmed as an early sign of SHOX-D [25], which loses significance with advancing bone age. Other early indicators of SHOX-D were ulnar tilt and enlarged radius diaphysis, frequently associated with radial bowing, while in the group of subjects with higher bone age triangularization of the distal radial epiphysis and ulnar tilt have been evidenced. The presence of an enlarged radius shaft was previously detected with quantitative peripheral CT in patients with SHOX-D by Soucek et al. [26], but is generally not considered in the evaluation of forearm radiographs. Regarding the carpal angle, an indicator of the pyramidization of the distal carpal row, the measurement was possible in females and males with bone age > 7 and > 8.6 years, respectively, without significant differences between SHOX-D + and SHOX-D -, with lower values in females. Pyramidalization is usually evident in LWD subjects [6], where much lower values of the angle were reported [18], but should be absent in the milder forms of SHOX-D.

Gender stratification revealed that radiological markers were significant only in females, who are usually more severely affected, with further worsening of stature and skeletal defects with puberty [27, 28]. In both sexes, the haploinsufficiency of the gene can be masked by a high parental height [28] and the main features of mesomelic disproportion of the limbs and Madelung deformity could only manifest themselves during the second decade of life [5] with an additional loss of height due to the reduction of the pubertal spurt [28, 29].

The high prevalence of SHOX-D in our large series of subjects with impaired growth highlights the importance of suspecting SHOX-D even in forms with mild phenotype, based on both clinical and radiological criteria. An early diagnosis of SHOX-D allows to start rhGH treatment early with an improvement on final height. The known limits of the Rappold scoring system [4], i.e. a low positive predictive value for the cutoff of 4 and a low sensitivity for that of 7, were also confirmed in our series, in which a score > 4 was found in 61.5% and > 7 in 30.8% of SHOX-D subjects. As for radiological signs, the hand/wrist X-ray is classically required in routine clinical practice for bone age assessment, as it is part of the diagnostic pathway of growth impairment; it should be read not only by radiologists but also by clinicians to assess the presence of the specific signs of SHOX-D. The addition of forearm X-ray assessment doubter provides additional support in case of suspected SHOX-D.

This study reinforces the notion that accurate auxological assessment not only of patients, but also of their parents, along with radiological analysis, both focused on detecting distinctive signs of SHOX-D, represent powerful screening tools for SHOX-D.

## Conclusions

**Fig. 2:**
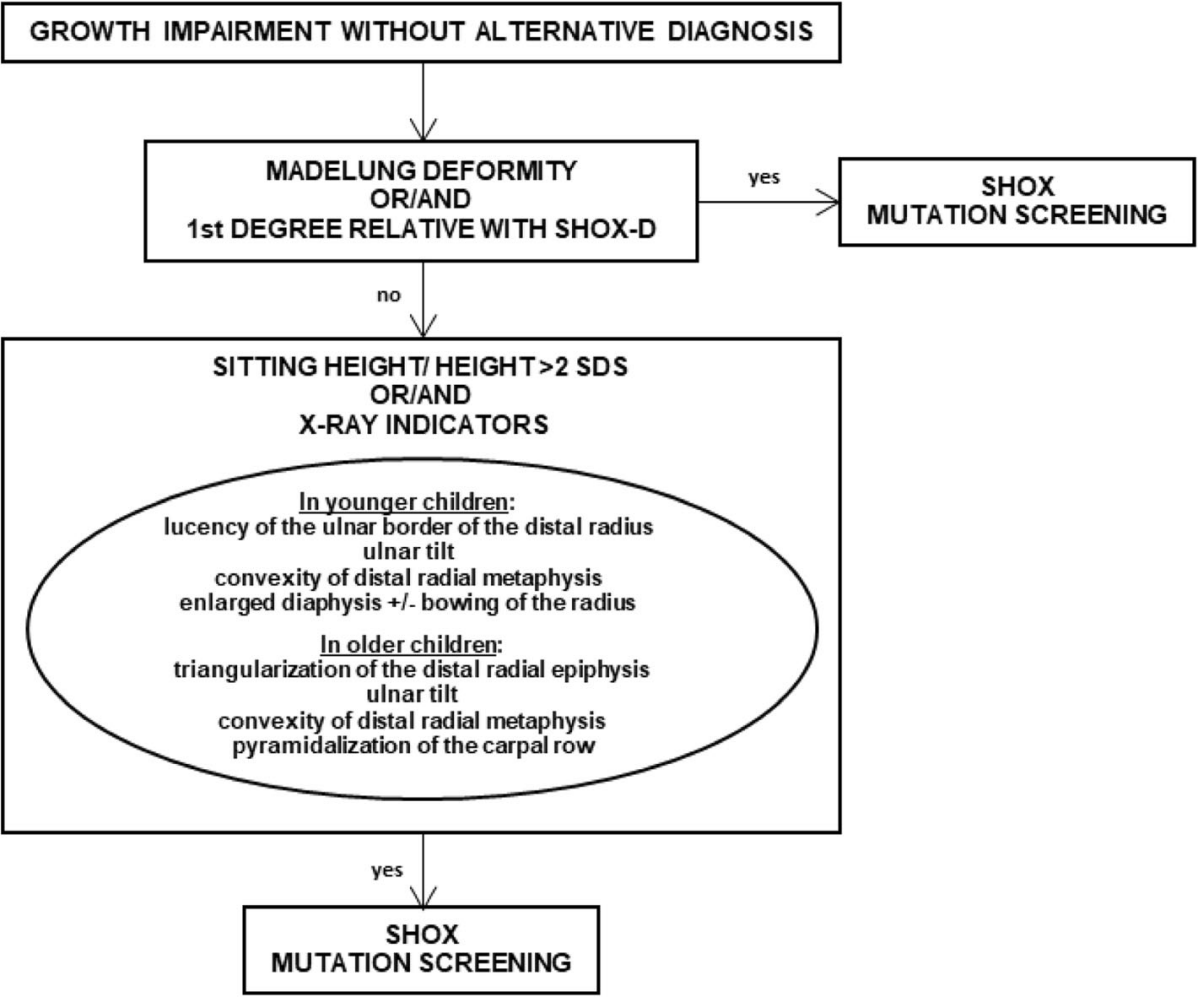
Diagnostic algorithm for SHOX mutation screening in children with growth impairment, obtained integrating literature data [5] and results of our study. Note: The absence of the reported signs does not rule out the diagnosis of SHOX deficiency, especially in very young children

Since SHOX-D can manifest with mild signs, the availability of a broader range of clinical and radiological criteria could help clinicians to select subjects for SHOX mutation screening. The auxological evaluation of children with growth impairment is often performed incompletely, while investigation of skeletal disproportions and X-ray signs is crucial, as is the auxological evaluation of parents. In this study, a new radiological sign, i.e. the convexity of the distal radial metaphysis, was proposed as an additional early radiological indicator of SHOX-D; our analysis also highlighted ulnar border lucency of the distal radius, ulnar tilt, and enlarged radius diaphysis as the most predictive signs of SHOX-D in younger children, particularly in females. Finally, a flow chart based on clinical- radiological criteria (Fig. 2) is proposed to facilitate clinicians in identifying patients to be subjected to genetic analysis for SHOX-D suspicion.

## Data Availability

All the data were obtained from the Auxological Center visit reports and from X-rays performed at the Radiology Unit of Regina Margherita Children’s Hospital in Turin. All data generated during this study are included in this article.

## Abbreviations

SHOX: Short-stature homeobox
SHOX-D: SHOX deficiency
LWD: Léri–Weill dyschondrosteosis
ISS: Idiopatic short stature
TI: Triangularization index

## References

[1] Marchini A, Ogata T, Rappold GA (2016). A track record on SHOX: from basic research to complex models and therapy. Endocr Rev.

[2] Rao E, Weiss B, Fukami M, Rump A, Niesler B, Mertz A, Muroya K, Binder G, Kirsch S, Winkelmann M, Nordsiek G, Heinrich U, Breuning MH, Ranke MB, Rosenthal A, Ogata T, Rappold GA (1997). Pseudoautosomal deletions encompassing a novel homeobox gene cause growth failure in idiopathic short stature and turner syndrome. Nat Genet.

[3] Marchini A, Marttila T, Winter A, Caldeira S, Malanchi I, Blaschke RJ, Häcker B, Rao E, Karperien M, Wit JM, Richter W, Tommasino M, Rappold GA (2004). The short stature homeodomain protein SHOX induces cellular growth arrest and apoptosis and is expressed in human growth plate chondrocytes. J Biol Chem.

[4] Rappold G, Blum WF, Shavrikova EP, Crowe BJ, Roeth R, Quigley CA, Ross JL, Niesler B. Genotypes and phenotypes in children with short stature: clinical indicators of SHOX haploinsufficiency. J Med Genet. 2007;44:306–13. 10.1136/jmg.2006.046581 PMC2597980 17182655

[5] Binder G (2011). Short stature due to SHOX deficiency: genotype, phenotype, and therapy. Horm Res Paediatr.

[6] Langer LO (1965). Dyschondrosteosis, a hereditable bone dysplasia with characteristic roentgenographic features. Am J Roentgenol Radium Therapy, Nucl Med.

[7] Child CJ, Kalifa G, Jones C, Ross JL, Rappold GA, Quigley CA, Zimmermann AG, Garding G, Cutler GB, Blum WF (2015). Radiological features in patients with short stature Homeobox-containing (SHOX) gene deficiency and turner syndrome before and after 2 years of GH treatment. Horm Res Paediatr..

[8] Gahunia HK, Babyn PS, Kirsch S, Mendoza-Londono R (2009). Imaging of SHOX-associated anomalies. Semin Musculoskelet Radiol.

[9] Blum WF, Crowe BJ, Quigley CA, Jung H, Cao D, Ross JL, Braun L, Rappold G, SHOX Study Group (2007). Growth hormone is effective in treatment of short stature associated with short stature homeobox-containing gene deficiency: two-year results of a randomized, controlled, multicenter trial. J Clin Endocrinol Metab.

[10] Blum WF, Ross JL, Zimmermann AG, Quigley CA, Child CJ, Kalifa G, Deal C, Drop SL, Rappold G, Cutler GB (2013). GH treatment to final height produces similar height gains in patients with SHOX deficiency and turner syndrome: results of a multicenter trial. J Clin Endocrinol Metab.

[11] Cameron N (1984). The measurement of human growth.

[12] Tanner JM, Goldstein H, Whitehouse RH (1970). Standards for children's height at ages 2-9 years allowing for heights of parents. Arch Dis Child.

[13] Tanner JM, Whitehouse RH, Takaishi M (1966). Standards from birth to maturity for height, weight, height velocity, and weight velocity: British children, 1965. I Arch Dis Child.

[14] Tanner JM, Whitehouse RH, Takaishi M (1966). Standards from birth to maturity for height, weight, height velocity, and weight velocity: British children, 1965. II Arch Dis Child.

[15] Fredriks AM, van Buuren S, van Heel WJ, Dijkman-Neerincx RH, Verloove-Vanhorick SP, Wit JM (2005). Nationwide age references for sitting height, leg length, and sitting height/height ratio, and their diagnostic value for disproportionate growth disorders. Arch Dis Child.

[16] Cacciari E, Milani S, Balsamo A, Spada E, Bona G, Cavallo L, Cerutti F, Gargantini L, Greggio N, Tonini G, Cicognani A (2006). Italian cross-sectional growth charts for height, weight and BMI (2 to 20 Yr). J Endocrinol Investig.

[17] Bertino E, Di Nicola P, Varalda A, Occhi L, Giuliani F, Coscia A (2012). Neonatal growth charts. J Matern Fetal Neonatal Med.

[18] Tauber M, Lounis N, Coulet J, Baunin C, Cahuzac JP, Rochiccioli P (2004). Wrist anomalies in turner syndrome compared with Leri-Weill dyschondrosteosis: a new feature in turner syndrome. Eur J Pediatr.

[19] McCarroll HR, James MA, Newmeyer WL, Manske PR (2010). Madelung's deformity: diagnostic thresholds of radiographic measurements. J Hand Surg Am.

[20] Tanner JM, Whitehouse RH, Cameron N, Marshall WA, Healy MJ, Goldstein H (1983). Assessment of skeletal maturity and prediction of adult height (TW2 method).

[21] Richards S, Aziz N, Bale S, Bick D, Das S, Gastier-Foster J, Grody WW, Hegde M, Lyon E, Spector E, Voelkerding K, Rehm HL (2015). Standards and guidelines for the interpretation of sequence variants: a joint consensus recommendation of the American College of Medical Genetics and Genomics and the Association for Molecular Pathology. Genet Med.

[22] Jorge AA, Souza SC, Nishi MY, Billerbeck AE, Libório DC, Kim CA, Arnhold IJ, Mendonca BB (2007). SHOX mutations in idiopathic short stature and Leri-Weill dyschondrosteosis: frequency and phenotypic variability. Clin Endocrinol.

[23] Munns CJ, Haase HR, Crowther LM, Hayes MT, Blaschke R, Rappold G, Glass IA, Batch JA (2004). Expression of SHOX in human fetal and childhood growth plate. J Clin Endocrinol Metab.

[24] Munns CF, Glass IA, LaBrom R, Hayes M, Flanagan S, Berry M, Hyland VJ, Batch JA, Philips GE, Vickers D (2001). Histopathological analysis of Leri-Weill dyschondrosteosis: disordered growth plate. Hand Surg.

[25] Binder G, Ranke MB, Martin DD (2003). Auxology is a valuable instrument for the clinical diagnosis of SHOX haploinsufficiency in school-age children with unexplained short stature. J Clin Endocrinol Metab.

[26] Soucek O, Zapletalova J, Zemkova D, Snajderova M, Novotna D, Hirschfeldova K, Plasilova I, Kolouskova S, Rocek M, Hlavka Z, Lebl J, Sumnik Z (2013). Prepubertal girls with turner syndrome and children with isolated SHOX deficiency have similar bone geometry at the radius. J Clin Endocrinol Metab.

[27] Fukami M, Nishi Y, Hasegawa Y, Miyoshi Y, Okabe T, Haga N, Nagai T, Tanaka T, Ogata T (2004). Statural growth in 31 Japanese patients with SHOX haploinsufficiency: support for a disadvantageous effect of gonadal estrogens. Endocr J.

[28] Kosho T, Muroya K, Nagai T, Fujimoto M, Yokoya S, Sakamoto H, Hirano T, Terasaki H, Ohashi H, Nishimura G, Sato S, Matsuo N, Ogata T (1999). Skeletal features and growth patterns in 14 patients with haploinsufficiency of SHOX: implications for the development of turner syndrome. J Clin Endocrinol Metab.

[29] Scalco RC, Melo SS, Pugliese-Pires PN, Funari MF, Nishi MY, Arnhold IJ, Mendonca BB, Jorge AA (2010). Effectiveness of the combined recombinant human growth hormone and gonadotropin-releasing hormone analog therapy in pubertal patients with short stature due to SHOX deficiency. J Clin Endocrinol Metab.

